# Physicochemical Characterization of the Oral Biotherapeutic Drug IMUNOR^®^

**DOI:** 10.3390/ph17091114

**Published:** 2024-08-23

**Authors:** Jitka Mucksová, Gabriela Borošová, Miloš Blazsek, Jiří Kalina, Lucie Minaříková, Zdeňka Svobodová

**Affiliations:** 1 BIOPHARM, Research Institute of Biopharmacy and Veterinary Drugs, Pohoří 90, 254 01 Chotouň, Czech Republic; jiri.kalina@bri.cz (J.K.); lucie.minarikova@bri.cz (L.M.); 2 ImunomedicA a.s., Chuderov118, 400 02 Ústí nad Labem, Czech Republic; gabriela.borosova@sdpharma.cz (G.B.); svobodova@imunomedica.cz (Z.S.); 3 Hameln rds s.r.o., Horná 36, 900 01 Modra, Slovakia; m.blazsek@hameln-rds.sk

**Keywords:** IMUNOR, quality specifications, SDS-PAGE, SE HPLC, RP UHPLC, amino acid analysis

## Abstract

IMUNOR is an oral biotherapeutic drug that had been developed, registered, and approved in 1997 in the Czech Republic and Slovakia. IMUNOR is a dialyzable leukocyte extract (DLE) prepared from swine leukocytes. It is characterized as a mixture of small peptides with molecular weights smaller than 12 kDa and a specific portion of nucleotides. The medical uses of IMUNOR include therapeutic applications within its registered range of indications, primarily for the treatment of immunodeficiencies, allergies, and certain acute or relapsing bacterial infections in adults and children. Despite the long-term clinical application of DLE, with strong evidence of positive therapeutic effects and no serious side effects, a detailed physicochemical specification of this mixture was lacking. We developed several methods for more in-depth physicochemical characterization of IMUNOR, including a spectrophotometric method for quantification of the total protein concentration and total DNA concentration in a mixture, several chromatographic methods for identification of individual components present in significant concentrations in IMUNOR, such as HPLC methods and the Sodium Dodecyl Sulphate Polyacrylamide Gel Electrophoresis method, and characterization of amino acid composition of this mixture. For the investigation of the variability among different batches of IMUNOR, five to nine representative batches from a standard manufacturing process on an industrial scale were utilized. Using the analytical methods, we verified and confirmed the batch-to-batch reproducibility of the biological product IMUNOR.

## 1. Introduction

Dialyzable leukocyte extract (DLE) is a mixture of peptides with molecular weights bellow 12,000 Daltons obtained from healthy human or porcine donors [1]. In 1992, Kirkpatrick described and characterized the term “transfer factor” (TF) at the molecular level [2]. The transfer factor, which is a part of the dialyzable leukocyte extract (DLE), has been used for the treatment of viral and bacterial infections, as well as an auxiliary treatment for some oncological diagnoses and autoimmune diseases, where the effective dosage was tested in numerous clinical trials [3,4]. Despite the long-term clinical application of DLE with strong evidence of positive therapeutic effects without any serious side effects, detailed physicochemical specification of this mixture was missing, and the molecular mechanism responsible for the DLE therapeutic effect is still under investigation [5]. Due to the complexity of the DLE mixture, several methods identifying the individual components have been tested and validated. Additionally, the batch-to-batch reproducibility of this biological drug product was verified and established [4,5,6,7,8,9,10]. The size-exclusion HPLC method revealed individual peaks corresponding to DLE’s components and their particular molecular weights were determined. A further effort was dedicated to the identification of individual components and evaluation of their relevance in terms of their immunomodulatory properties via sequence analysis followed by proteomic characterization [11,12].

Several publications provided evidence that transfer factors can transfer cell-mediated immunity from the immunized donor to a naïve recipient even across different species [2]. Various types of transfer factor derived from different mammalian sources are applied in clinical settings. According to published scientific data, a considerable effort has been made to determine the structure and composition of DLE, and to obtain the transfer factor that is prepared from leukocytes acquired from various mammalian species, including porcine, bovine, and rabbit spleen-derived DLE, along with its immunoregulatory properties and efficacy [13,14,15,16,17,18].

In 1997, a biotherapeutic drug product denominated “IMUNOR Lyophilised Oral Solution” received approval from Czech and Slovak health authorities for therapeutic applications according to the registration range of indications, mainly for the treatment of immunodeficiencies, allergies, and some acute or relapsing bacterial infections in adults and children [19,20]. Despite the positive effect of IMUNOR, which was confirmed by numerous clinical studies evaluating its therapeutic effect [20,21,22,23,24], and its long-term clinical application, which proved its efficacy as well as its safety ensured by pharmacovigilance tracking according to the valid legislation, a comprehensive physicochemical characterization due to its highly complex composition is necessary [9,10]. The development of multiple analytical methods is essential for the qualitative specification of the IMUNOR drug product consisting of a complex mixture of peptides and nucleotides. The physicochemical characterization of biological products is a crucial step in determination of their composition to establish the critical qualitative characteristics, as well as the consistency among production batches, which are the essential attributes to ensure the identity and safety of a biological drug product [25].

The aim of this study was to describe and summarize the physicochemical properties of IMUNOR batches containing DLE having molecular weight below 12,000 Daltons (Da) as an active pharmaceutical ingredient. We present the results achieved by an application of several techniques used for the physicochemical specification of IMUNOR, including evaluation of the reproducibility of these parameters among randomly selected production batches.

The IMUNOR batches were characterized by a spectrophotometric method that allowed quantification of the total protein content and total DNA content, and, furthermore, their evaluation by liquid chromatography separation techniques such as reversed-phase (RP) chromatography, size-exclusion (SE) chromatography and SDS PAGE electrophoresis. Additionally, the amino acid (AA) composition of IMUNOR samples was determined by pre-column derivatization of the peptide hydrolysate.

## 2. Results

### 2.1. Leukocytes

In a quantitative analysis of leukocyte counts in 100 porcine blood samples, which serve as the raw material for preparation of IMUNOR™ Substance, each sample was measured twice. The average measured value was 18.72 × 10^6^ leukocytes per milliliter of swine blood. This value is consistent with published data [26,27].

### 2.2. Protein Quantification

The nine batches of IMUNOR Substance were tested by the BCA (bicinchoninic acid) method in three independent measurements with regard to the protein content. Due to the expected hydrophilic character of the tested IMUNOR Substance preparation (based on the results of the identity test according to thin-layer chromatography (TLC), typical profile), the BCA method was selected to determine the protein content. The theoretical value of the protein content is 0.40 mg/mL [5]. 

**Figure 1:**
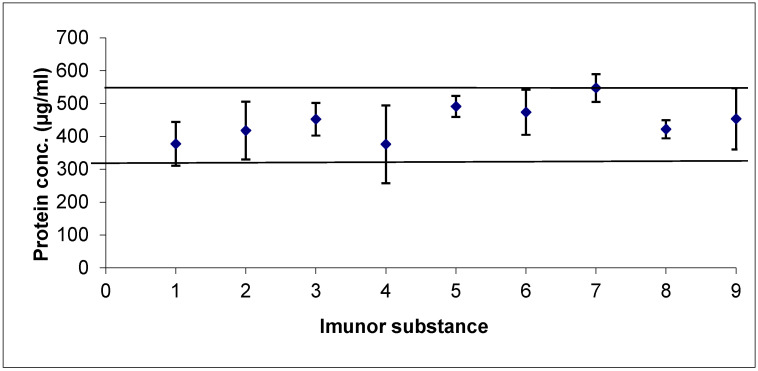
Analysis of the protein content among IMUNOR batches stained using the BCA method. Blue diamonds and ± SD intervals are shown for each batch. The variation among the batches was in a ± 2 SD interval (solid lines).

The average protein content determined in the nine batches of IMUNOR Substance was 0.446 mg/mL (+/−54.9 mg/mL), which is within the expected range, and was expressed as 111.5% of the theoretical expected value. The variation among the batches was in a +/−2 SD interval, which proves the manufacturing process consistency (Figure 1).

### 2.3. DNA Quantification

**Figure 2:**
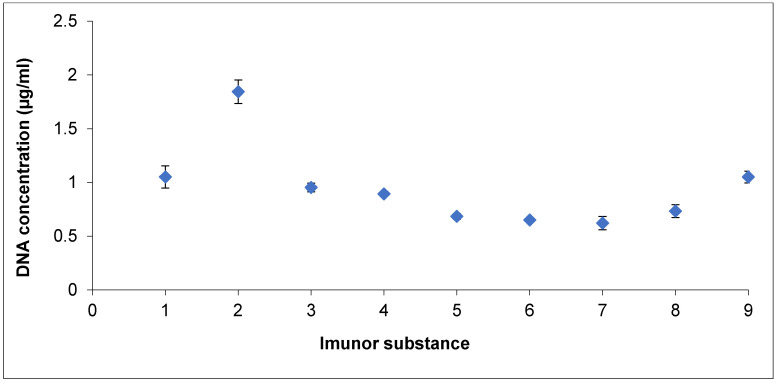
Analysis of DNA content among IMUNOR batches by the fluorometric method. Blue diamonds and ± SD intervals are shown for each batch. The variation among batches was in a ±3 SD interval (solid lines).

IMUNOR Substance is specified as a mixture of peptides and nucleotides currently characterized as low-molecular-weight substances present in a mixture in a ratio higher than 1.8, as determined spectrophotometrically at A260/A280. In addition to determining the total protein concentration (as described above), we tried to verify methods for nucleotide concentration determination [28]. The content of DNA was determined by the fluorometric method, yielding values ranging from 0.571 µg/mL to 1.950 µg/mL, with an average value of 0.942 µg/mL. The variation among the batches was within a ± 3 SD range (Figure 2). The total RNA in the tested IMUNOR Substance samples (IM1–IM9) was not detected.

### 2.4. SDS-PAGE Profile

**Figure 3:**
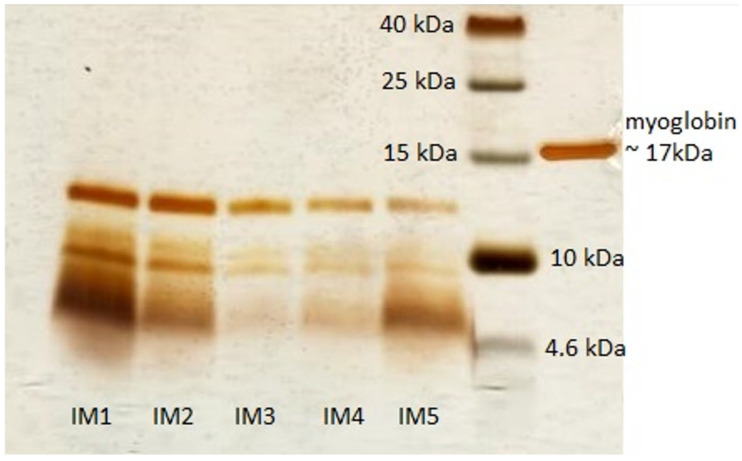
Electrophoretic profile of five IMUNOR batches IM1-IM5 by SDS PAGE under denaturing conditions. Electrophoresis was performed in 16% polyacrylamide gel using a Tris-tricine system; protein standard (1.7–40 kDa) was used as a molecular weight marker. Equine myoglobin (1.25 µg) was used as a control. Bands were detected by silver stain.

We performed SDS-PAGE electrophoresis to determine the typical peptide profile of IMUNOR Substance. The electrophoretic profile of the peptide components of IMUNOR Substance was determined under non-denaturing and denaturing conditions. Five batches from the regular manufacturing process (IM1—001000122; IM2—002000322; IM3—003000522; IM4—004000522; IM5—005000822) were evaluated to verify the peptide pattern and consistency among these batches. The molecular weight of individual components was determined and compared. A similar peptide profile was observed among the five batches, with four bands representing molecules of approximately 11.5 kDa, 9 kDa, 8 kDa, and 6.5 kDa (Figure 3). The SDS-PAGE method used in this analysis, however, may not be sensitive enough to detect the smaller peptide components of IMUNOR due to its low resolution and sensitivity.

### 2.5. Reversed-Phase (RP) UHPLC

RP-UHPLC analyses of five IMUNOR batches representing porcine DLE revealed a highly similar chromatographic profile among the tested samples. In total, twelve chromatographic peaks were detected in the analyzed batches at 214 nm, all eluted within 11 min. All peaks were well separated with resolutions above 3.0, except for the earliest eluted peak pair with the lowest capacity factor (k) ranging from 0.3 to 0.4 and the lowest resolution (Rs = 1.1). 

**Figure 4:**
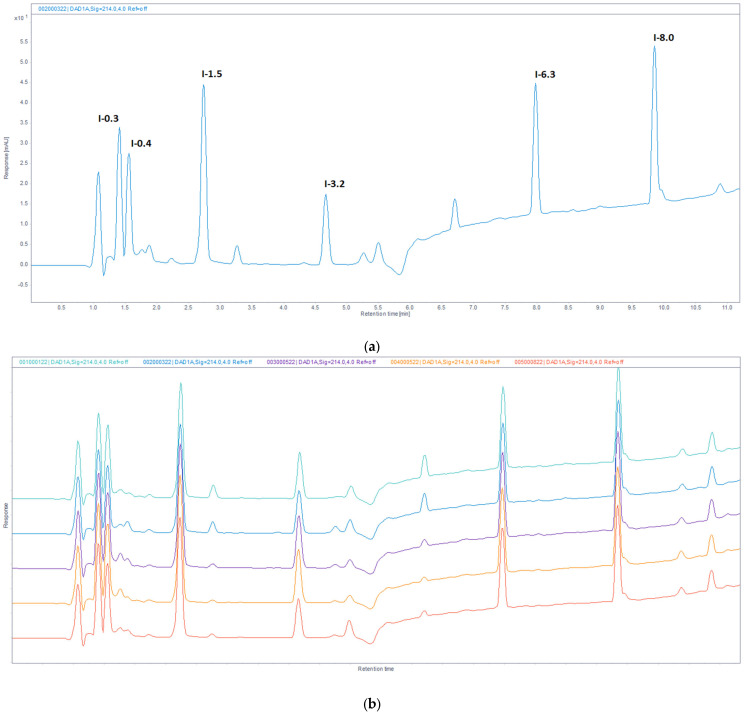
(**a**). Reversed-phase chromatographic profile of IMUNOR sample IM2 at 214 nm with identified six major peaks designated with respective capacity factor numbers of I-0.3, I-0.4, I-1.5, I-3.2, I-6.3, and I-8.0. The IMUNOR sample was analyzed on an Acquity UPLC Peptide BEH C18, 300 Å 1.7 µm (2.1 × 150 mm) column employing gradient elution of 0.1% TFA aqueous solution and 0.1% TFA in acetonitrile aqueous solution (3:7, *v*/*v*) as mobile phases. The flow rate was maintained at 0.4 mL/min, column temperature was kept at 35 °C, and detection was performed by a DAD detector in the range from 200 to 400 nm. The peak area percentage was calculated using the OpenLab chromatography data system. (**b**). Overlay of reversed-phase chromatographic profiles of IMUNOR batches IM1, IM2, IM3, IM4, and IM5, arranged from top to bottom and detected at 214 nm. IMUNOR batches were analyzed on an Acquity UPLC Peptide BEH C18, 300 Å 1.7 µm (2.1 × 150 mm) column with gradient elution of 0.1% TFA aqueous solution and 0.1% TFA in acetonitrile aqueous solution (3:7, *v*/*v*) as mobile phases. The flow rate was maintained at 0.4 mL/min, the column temperature was kept at 35 °C, and detection was performed by a DAD detector in the range from 200 to 400 nm. The peak area percentage was calculated by the OpenLab chromatography data system.

**Figure 5:**
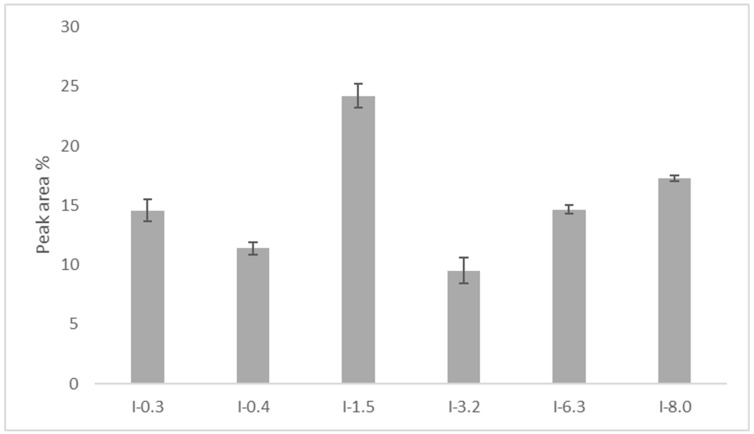
Average area percentage of six major peaks I-0.3, I-0.4, I-1.5, I-3.2, I-6.3, and I-8.0 determined in five IMUNOR batches IM1, IM2, IM3, IM4, and IM5.

Six major peaks, representing more than 9% of the total peak area, were consistently observed in each tested sample and designated as I-0.3, I-0.4, I-1.5, I-3.2, I-6.3, and I-8.0 according to their capacity factor numbers (Figure 4a). The analysis of five IMUNOR samples IM1–IM5 determined the percentage area of major IMUNOR components, which ranged from 8% to 25%, as shown in Figure 4b. Additional minor peaks designated as I-0.6, I-2.0, I-3.8, I-4.0, I-5.0, and I-8.9 were also identified in IMUNOR batches, with their total content varying from 6% to 12% (Figure 5).

UV spectra of the detected peaks were examined by a DAD detector. Significant differences in absorbance maxima and absorbance intensity of individual peaks were observed within the range of 240 nm–300 nm. The detected IMUNOR compounds were classified into four groups with their absorbance maxima at about 280 nm for I-3.2, I-4.0, I-8.0, and I-8.9, about 270 nm for I-0.6 and I-5.0, about 260 nm for I-0.3, I-1.5 I-2.0, and I-6.3, and about 250 nm for I-0.4 and I-3.8. 

### 2.6. Size-Exclusion (SE) UHPLC

**Figure 6:**
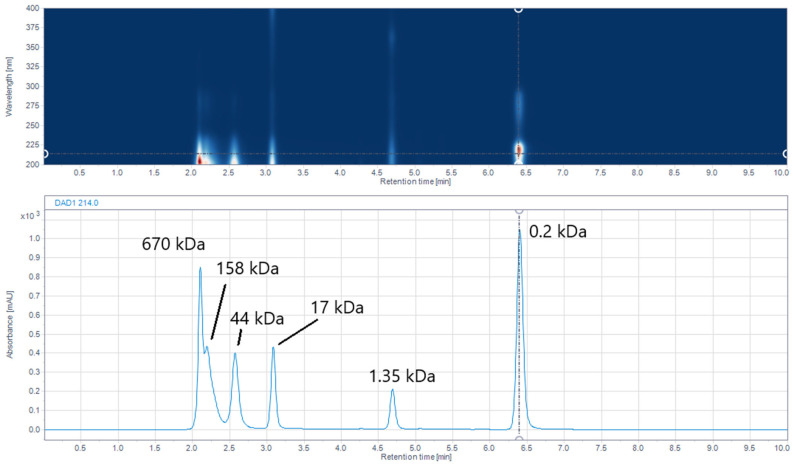
Diode array detector (DAD) chromatogram of the standard mixture shown as an iso-absorbance plot over 200–400 nm (upper blue area) and the respective chromatogram at 214 nm (lower blue line) with indicated molecular weights of standard mixture components: thyroglobulin (670 kDa), bovine γ-globulin (158 kDa), chicken ovalbumin (44 kDa), equine myoglobin (17 kDa), vitamin B12 (1.35 kDa), and tryptophane (0.2 kDa) indicated by a dotted line. The standard mixture was analyzed on an Acquity UPLC Peptide BEH SEC, 125 Å 1.7 µm (4.6 × 150 mm) column with 100 mM phosphate buffer pH 7.0 as the mobile phase. The flow rate was maintained at 0.4 mL/min, the column temperature was kept at 35 °C, and detection was performed by a DAD detector in the range from 200 to 400 nm.

**Figure 7:**
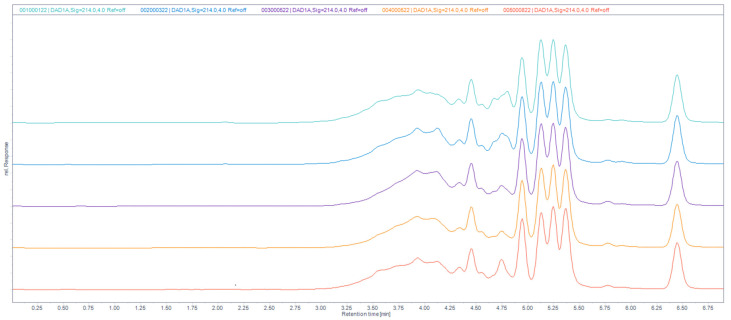
Overlay of size-exclusion chromatographic profiles of IMUNOR samples IM1, IM2, IM3. IM4, and IM5, in order from top to bottom, at 214 nm. IMUNOR samples were analyzed on an Acquity UPLC Peptide BEH SEC, 125 Å 1.7 µm (4.6 × 150 mm) column with 100 mM phosphate buffer pH 7.0 as the mobile phase. The flow rate was maintained at 0.4 mL/min, the column temperature was kept at 35 °C, and detection was performed by a DAD detector in the range from 200 to 400 nm.

SE chromatography was employed to determine the molecular weights of IMUNOR components using a UHPLC calibrated column and GPC/SEC Software. The column was calibrated with a standard mixture containing thyroglobulin, bovine γ-globulin, chicken ovalbumin, equine myoglobin, vitamin B12, and tryptophane (Figure 6). In the analysis of the five IMUNOR batches IM1 to IM5, comparable chromatographic profiles were observed, although poor separation between the main components was achieved, as presented in Figure 7.

**Figure 8:**
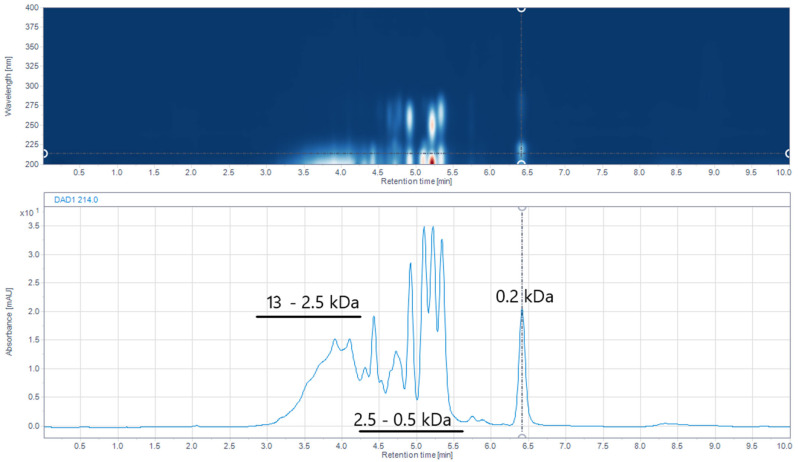
Size-exclusion chromatographic profile of IMUNOR sample IM2 presented as an iso-absorbance DAD plot in the range 200–400 nm (upper blue area) and respective chromatogram at 214 nm (lower blue line) with indicated molecular weight ranges of 18–2.5 kDa, 2.5–0.5 kDa, and 0.2 kDa indicated by a dotted line. The IMUNOR sample was analyzed on an Acquity UPLC Peptide BEH SEC, 125 Å 1.7 µm (4.6 × 150 mm) column with 100 mM phosphate buffer pH 7.0 as the mobile phase. The flow rate was maintained at 0.4 mL/min, the column temperature was kept at 35 °C, and detection was performed by a DAD detector in the range from 200 to 400 nm. Molecular weights of IMUNOR components were calculated using GPC/SEC Software (version 1.3) for the OpenLab chromatography data system employing a narrow standard calibration.

**Figure 9:**
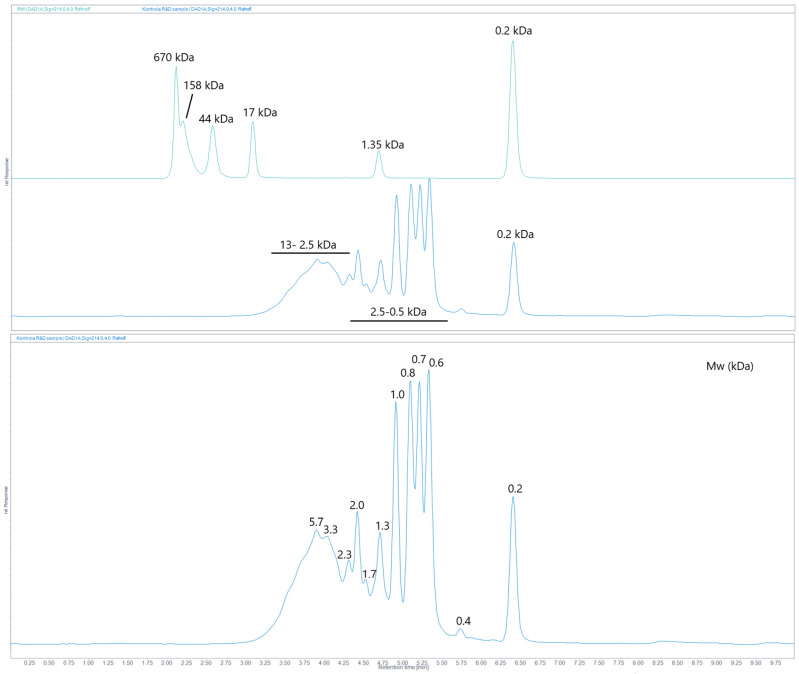
The upper figure represents an overlay of chromatograms of the standard mixture (upper green line) and IMUNOR sample (lower blue line) detected at 214 nm with indicated molecular weights of standard components and determined molecular weight regions of components in the IMUNOR batch, separation in SE mode. The lower chromatogram provides the depicted determined molecular weights (kDa) of detected components.

Nevertheless, the determination of molecular weight ranges of IMUNOR components was feasible and all were found to be within 0.1 kDa to 13 kDa. These values are consistent with the IMUNOR ultrafiltration production step, where a UF membrane with porosity of 10 kDa is employed. As shown in Figure 8, IMUNOR components of largest molecular weights (2.5–13 kDa) were eluted in the form of a broader peak with an apex of about 5.7 kDa. The molecular weights of the main components were found to be distributed within a relatively narrow range of 0.5 kDa to 2.5 kDa, while the molecular weight of the last eluting component was determined to be 0.2 kDa (Figure 9). 

**Figure 10:**
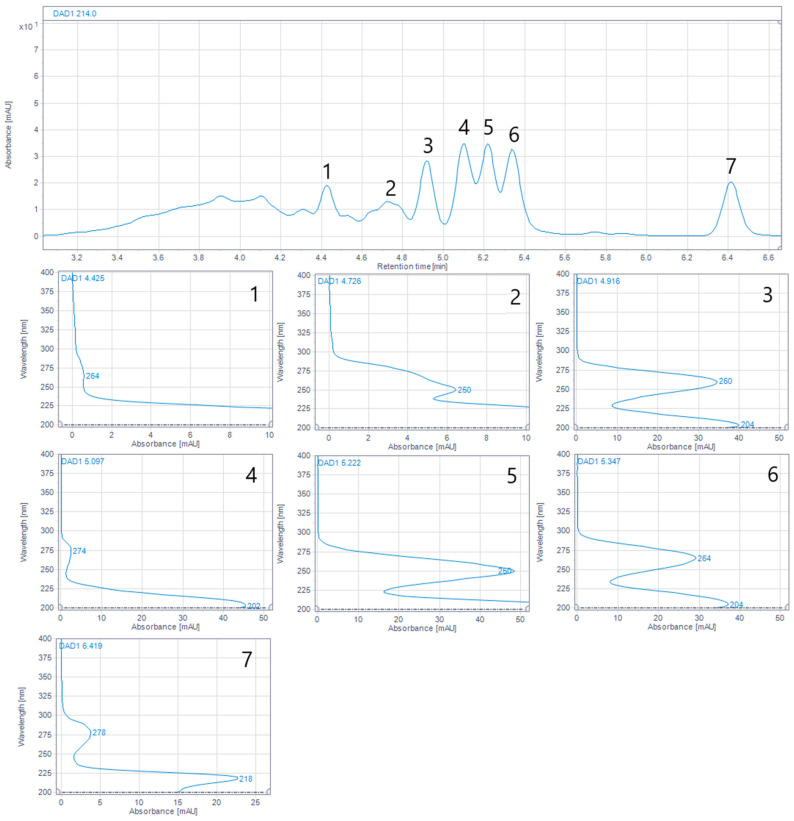
Magnified area of the chromatogram of IMUNOR batch IM2 (chromatogram range between 3.1 and 6.6 min) with corresponding UV spectra of selected peaks within the molecular weight range of 2.5–0.2 kDa. Determined wavelengths of absorption maxima are numerically presented (blue numbers) in respective UV spectrums. The sample was analyzed on an Acquity UPLC Peptide BEH SEC, 125 Å 1.7 µm (4.6 × 150 mm) column with 100 mM phosphate buffer pH 7.0 as the mobile phase. The flow rate was maintained at 0.4 mL/min, the column temperature was kept at 35 °C, and detection was performed by a DAD detector in the range from 200 to 400 nm.

Based on the above analysis, the specific molecular weights of some peptide components of IMUNOR were identified with corresponding molar masses ranging from 0.2 kDa to 5.7 kDa. Although no baseline separation of peaks relative to the main components was achieved, seven characteristic peaks, examined in all analyzed IMUNOR batches IM1 to IM5, were defined, and their UV spectra were examined by a DAD detector (Figure 10). The found UV characteristics of the peaks were in good correlation with the UV characteristics of the peaks determined by RP HPLC.

### 2.7. Amino Acid Analysis

**Figure 11:**
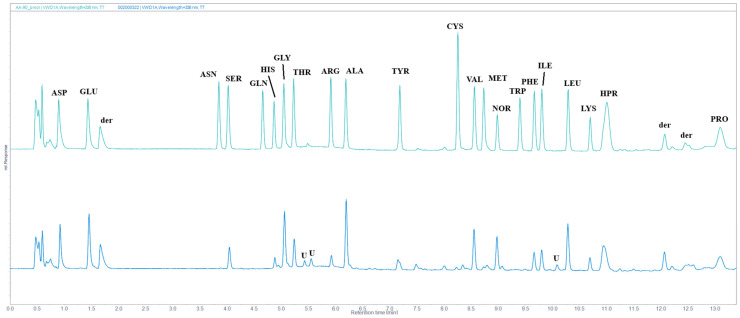
Comparison of chromatographic profiles of amino acid standard solution (green line) and IMUNOR sample IM2 (blue line) with designated amino acid peaks: ASP—Aspartic acid, GLU—Glutamic acid, ASN—Asparagine, SER—Serine, GLN—Glutamine, HIS—Histidine, GLY—Glycine, THR—Threonine, ARG—Arginine, ALA—Alanine, TYR—Tyrosine, CYS—Cystine, VAL—Valine, MET—Methionine, NOR—Norvaline (internal standard), TRP—Tryptophan, PHE—Phenylalanine, ILE—Isoleucine, LEU—Leucine, LYS—Lysine, HPR—Hydroxyproline, PRO—Proline. Peaks due to derivatization agents are labeled as “der”, while unknown peaks in the IMUNOR sample are labeled as “U”. IMUNOR acidic hydrolysate was analyzed on an AdvanceBio AAA C18 HPLC column (2.7 µm 4.6 × 100 mm) coupled with an AdvanceBio AAA guard column (2.7 µm, 4.6 × 5 mm) employing gradient elution of 10 mM borate-phosphate buffer (pH 9.2) and acetonitrile:methanol:water mixture (45:45:10, v:v:v) at a flow rate of 1.5 mL/min. The temperature for the column was set to 40 °C. Separated amino acids were detected by a UV/VIS detector at 338 nm for OPA-derivatized primary amino acids and at 262 nm for FMOC-derivatized secondary amino acids.

**Figure 12:**
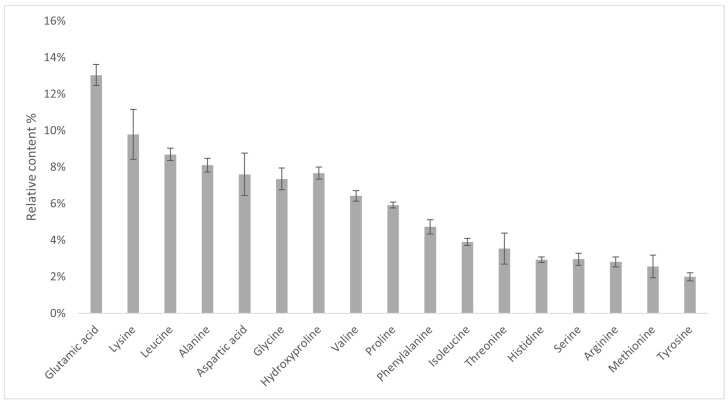
Average relative percentage content (m/m) of identified amino acids in five IMUNOR batches IM1, IM2, IM3, IM4, and IM5. Glu (13.06%), Lys (9.78%), and Leu (8.70%) are the most abundant amino acids in IMUNOR samples, whereas Met (%) and Tyr (%) are the least abundant. The determined total content of the identified proteinogenic amino acids was considered as 100% for abundance calculations. The content of individual amino acids was calculated specifically against amino acid standards and is expressed as a weight percentage in the tested IMUNOR.

**Figure 13:**
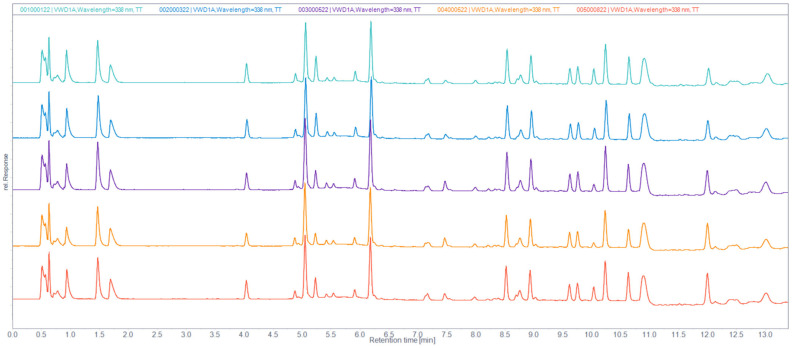
Comparison of chromatographic profiles of amino acids in IMUNOR batches IM1, IM2, IM3, IM4, and IM5, in order from top to bottom. IMUNOR acidic hydrolysates were analyzed on an AdvanceBio AAA C18 HPLC column (2.7 µm 4.6 × 100 mm) coupled with an AdvanceBio AAA guard column (2.7 µm, 4.6 × 5 mm) employing gradient elution of 10 mM borate-phosphate buffer (pH 9.2) and acetonitrile:methanol:water mixture (45:45:10, *v*:*v*:*v*) at a flow rate of 1.5 mL/min. The temperature for the column was set to 40 °C. Separated amino acids were detected by a UV/VIS detector at 338 nm for OPA-derivatized primary amino acids and at 262 nm for FMOC-derivatized secondary amino acids.

Amino acid analysis of IMUNOR acidic hydrolysates was used to determine the peptide composition of IMUNOR components. By comparing the chromatogram of the amino acid standard solution, 17 amino acids in total were identified in chromatograms of the analyzed IMUNOR acidic hydrolysates (Figure 11). Glutamic acid, lysine, leucine, alanine, aspartic acid, glycine, hydroxyproline, and valine were identified as the most abundant amino acids with individual relative content above 5% (Figure 12). Similar amino acid profiles among the five tested IMUNOR batches were observed concerning the identified amino acids (Figure 13) but also the content of three unidentified peaks presented in each IMUNOR batch (Figure 11). The amino acid composition profile among the five batches and the relative average abundance of the 17 proteinogenic amino acids were highly reproducible and consistent, with an RSD of less than 24%. 

## 3. Discussion

IMUNOR is an oral porcine DLE obtained from peripheral blood of healthy pig donors, which exhibits immunomodulatory properties in the treatment of infections, allergies, and autoimmune diseases [3,4,13,17,21,22,23], particularly when co-administered with the standard pharmacological treatment. The quality of IMUNOR is verified by a regular in-process control, a quality control under GMP conditions for its active substance (IMUNOR Substance), and a final quality control of the drug product “IMUNOR Lyophilised Solution”. 

Three HPLC separation analytical techniques were employed for the precise characterization of IMUNOR batches confirming their reproducible composition consisting of a mixture of several peptides. The molecular weights of IMUNOR components were determined using SE chromatography. IMUNOR components of largest molecular weights (2.5 kDa–13 kDa) were eluted as a non-distinguished broader peak with an apex of about 5.7 kDa. Specific molecular weights of all peptide components of IMUNOR were identified with corresponding molar masses in the range from 0.2 kDa to 5.7 kDa. Although baseline separation of peaks from the main components was not achieved, seven characteristic peaks were defined, and their UV spectra were examined by a DAD detector. 

Authors [5] developed an SE HPLC method that enabled them to distinguish eight typical peaks representing DLE from human origin defined as a complex mixture of low-molecular-weight peptides with molecular weights of around 7 kDa and a polydispersity index of around 1.1 ± 0.1. This product presents a typical 8-chromatographic-peak fingerprint with a molecular weight ranging from 0.2 kDa to 17 kDa, and the fifth most abundant peak (28.09/100%) showing an average retention time of 5.98 min [5]. Similarly, Cardoso et al. [9] evaluated a human DLE and they revealed eight elution peaks. IMUNOR porcine DLE also possesses a typical chromatographic fingerprint pattern, with seven typical peaks being present in all examined IMUNOR batches. The determined molecular weights of these components were highly reproducible over all tested batches, all ranging within the confidence interval of less than 0.01 kDa and having a narrow distribution with the mean polydispersity index of 1.0. The polydispersity index of the broadest peak found in all IMUNOR batches was about 1.13 ± 0.01 with an average retention time of 3.92 ± 0.3 min. 

Further development of the presented method is necessary, as the peptide spectrum range representing individual molecules with sizes 2.5–13 kDa has not been separated sufficiently and is presented as one non-distinguished peak.

Additionally, we complemented the results achieved by the SE chromatography with the SDS-PAGE electrophoretic method to identify individual peptide profiles in five different batches of IMUNOR. The electrophoretic evaluation was homogeneous across the analyzed batches, revealing a consistent peptide profile with four bands presenting molecules with similar molecular weights, i.e., 11.5 kDa, 9 kDa, 8 kDa, and 6.5 kDa. Due to the limited resolution and sensitivity of the SDS-PAGE method, smaller peptide components of IMUNOR were not detectable compared to SE chromatography. When comparing the results of the electrophoretic profile of human DLE [7] with two bands, we detected a typical four-band SDS-PAGE pattern in the case of porcine DLE IMUNOR Substance. This method has the potential to be used as a primary identity test in DLE specification.

The hydrophobic and hydrophilic characteristics of the main IMUNOR components were analyzed using reversed-phase chromatography, resulting in effective separation of the primary components. RP-UHPLC analyses of five IMUNOR batches revealed a highly consistent chromatographic profile across the tested samples. Six major peaks with peak area constituting more than 9% were observed in each tested sample, and the average percentage area of the primary IMUNOR components ranged from 8% to 25% with high reproducibility (RSD less than 10%). 

The authors of a previous study [7] presented data of peptide hydrophobicity analysis using the RP HPLC method for human DLE and they identified four main chromatographic peaks, which confirms that human DLE is a complex peptide mixture. The difference revealed between human and porcine DLE might be due to the different leukocyte origin and, but also very probably, due to a different process of its preparation, for instance, using a different method for leukocyte disintegration, in which concomitant proteolysis may differ, which can result in deeper fractionation of the present leukocyte proteins. 

Amino acid analysis of the IMUNOR samples exposed to an acidic hydrolysis revealed an average relative percentage (m/m) of glutamic acid (13.6%), lysine (9.78%), leucine (8.7%), alanine (8.12%), acid asparagine (7.6%), glycine (7.36%), hydroxyproline (7.66%), and valine (6.42%), indicating the most abundant proteinogenic amino acids in IMUNOR, while methionine and tyrosine were found to be the least abundant. Similar reversed-phase chromatographic studies were also performed with human DLE (4). However, it was observed that the representation of the most abundant amino acids from the peptide fraction of human DLE differs from that of the pig. The most prevalent amino acids were glycine (18.3%), glutamic acid, and alanine, while the least abundant were methionine and arginine. These differences may be caused by the different source of DLE, as DLE from the porcine spleen shows original amino acid composition where glutamic acid is most abundant as well [15,29]. Similar to human DLE, amino acid analysis of IMUNOR revealed three unidentified peaks in the amino acid chromatographic profile consistently across all batches tested. Identifying these non-proteinogenic amino acids will be the subject of further studies. 

All methods mentioned above have confirmed that IMUNOR is a complex composition of peptides and attached residues of nucleotides. Our SE HPLC analysis and SDS PAGE analysis both illustrate the wide variety of peptides present in the mixture. This will be subjected to further comprehensive analysis, and especially further modification and adjustment of our current SE HPLC method.

For comprehensive physicochemical characterization of the IMUNOR drug product, we have developed several analytical methods confirming that its manufacturing process is consistent and robust. All the techniques employed in this work demonstrated high reproducibility across the analyzed IMUNOR batches. Some of these methods will be utilized to establish the typical fingerprint of IMUNOR.

## 4. Materials and Methods

### 4.1. Analytical Samples

IMUNOR batches tested in this study were manufactured by company ImunomedicA (Ústí and Labem, Czech Republic), under Good Manufacturing Practice conditions using a specific robust manufacturing procedure according to the valid production method. IMUNOR, a porcine dialyzable leukocyte extract, was obtained from the peripheral blood of 6–7-month-old pigs, from a Landrase breed, and veterinary certified as healthy individuals. All animals originated from Czech farms. The raw material was obtained by separation of leukocytes from the fresh swine peripheral blood stabilized by an aqueous solution containing glucose monohydrate, citric acid monohydrate, and sodium citrate dihydrate in a ratio of 1: 0.3: 1. The received blood bank was immediately processed, initially by centrifugation to separate the leukocyte layer. This was then collected and frozen at −20 °C in plastic containers. Subsequently, the thawed leukocytes were subjected to ultrasonic disintegration followed by a repeated freezing and thawing procedure followed by ultrafiltration in a stirred cell with a membrane with a 10,000 Dalton cut-off. The collected ultrafiltrates were frozen again and the thawed material was then subjected to vacuum evaporation to reduce the final volume tenfold. The concentrated ultrafiltrate was tested for specified qualitative parameters and frozen again at −20 °C. The thawed ultrafiltrate was then filtered through a 0.2 µm filter to remove microbiological residues and immediately poured into lyophilization trays and placed into a lyophilization unit. The material was freeze-dried according to the manufacturer’s protocol.

The freeze-dried IMUNOR Substance was collected and stored in a sterile dark-glass container in a chillroom at 6–8 °C. This material was subjected to a quality control protocol according to criteria specified in the registration documentation approved by the Czech drug authority, which is the State Institute for Drug Control. These quality control characteristics include (a) measurement of biological activity of IMUNOR using splenocyte proliferation assay, (b) identity test according to TLC typical profile, (c) spectrophotometric ratio of low-molecular-weight substances, (d) water content not more than 10%, and (e) microbial suitability as per Ph.Eur. [30].

IMUNOR Substance samples were obtained from nine typical randomly selected production batches: IMUNOR 1—001000122 (IM1); IMUNOR 2—002000322 (IM2); IMUNOR 3—003000522 (IM3); IMUNOR 4—004000522 (IM4); IMUNOR 5—005000822 (IM5); IMUNOR 6—006001022 (IM6); IMUNOR 7—007001122 (IM7); IMUNOR 8—002001021 (IM8); IMUNOR 9—001000921 (IM9).

### 4.2. Porcine Leucocyte Quantification

The quantification of leukocytes was conducted over a period of five weeks, involving two collections of ten samples per week, totaling 100 samples of porcine blood. The commercially available separation medium Histopaque 1077 (Merck, Darmstadt, Germany) was used to separate blood samples using density gradient sedimentation to avoid unwanted cell lysis prior to counting [31]. Samples of non-clotting blood, treated with citrate solution, were diluted with PBS in a ratio of 1:1 and then loaded on Histopaque 1077 separation medium in a 2:1 ratio. After centrifugation at 300× *g* for 20 min and washing in PBS, isolated leukocytes were counted in a Bürker chamber.

### 4.3. Protein Quantification

The protein concentration was measured by the bicinchoninic acid (BCA) method using the PierceTMBCA kit (Thermo Fisher Scientific, Waltham, MA, USA) according to the manufacturer’s instructions. The bovine serum albumin solution was used as a standard for the calibration curve. Samples were analyzed in a MULTISKAN FC spectrophotometer (Thermo Fisher Scientific, Waltham, MA, USA), absorbance was measured at 562 nm, and the protein concentration was calculated from these values.

### 4.4. DNA or RNA Quantification

DNA or RNA concentration was measured by the fluorometric method [28] using the Qubit RNA Assay Kit and/or Qubit dsDNA BR Assay Kit (Thermo Fisher Scientific, Waltham, MA, USA) according to the manufacturer´s instructions by mixing the two components Qubit Buffer and Qubit Reagent for each kit separately at a 200:1 ratio, respectively. Standards for calibration curves were prepared by appropriate dilution of stock solutions (kit components). In parallel with the standards, tested batches (IM1 to IM9) of IMUNOR Substance of unknown concentration were prepared by appropriate dilution (0.2 mg/μL). The total volume of the reaction was 200 μL. The tubes were incubated for 2 min at room temperature. Then, using a Qubit 2.0 fluorimeter, the fluorescence intensity (RFU) of the standards was measured, and calibration curves for both DNA and RNA were constructed. The fluorescence intensity (RFU) of the unknown samples was subsequently recalculated to RNA or DNA concentrations by subtracting values from calibration curves taking into account the dilution factor (for each kit separately).

### 4.5. Sodium Dodecyl Sulphate Polyacrylamide Gel Electrophoresis (SDS-PAGE)

The electrophoretic profile of the peptide components of IMUNOR Substance was determined under denaturing and non-denaturing conditions [7]. IMUNOR Substance samples (10 mg sample in 50 µL demineralized water) were diluted in Novex^®^ Tricine SDS Sample buffer with/without reducing agent in ratio 1:1 (Thermo Fisher Scientific) and heated at 86 °C for 5 min. Then, 15 ul of each sample was loaded onto Novex TM 16% pre-packed Tricine polyacrylamide Gel (Thermo Fisher Scientific). We used equine myoglobin (17 kDa, Merck) and a protein standard (1.7–40 kDa, Spectra Low Range, Thermo SCIENTIFIC) as molecular weight markers. Electrophoresis was performed at 80 V for 2.5 h using a Novex^®^ Tris-Tricine SDS buffer system (Thermo Fisher Scientific, Waltham, MA, USA) and the separated peptides were visualized by silver nitrate staining (Pierce^TM^ Silver Stain Kit, Thermo Fisher Scientific, Waltham, MA, USA).

### 4.6. Reversed-Phase (RP) UHPLC

Reversed-phase ultra-performance liquid chromatography with gradient elution was employed to investigate the hydrophilic properties of IMUNOR components. IMUNOR Substance samples (10 mg sample in 2 mL purified water) were diluted in mobile phase in ratio 1:9 (*v*/*v*). Before injecting 4 μL of sample solution into the 1260 Infinity II UHPLC system (Agilent Technologies, Santa Clara, CA, USA), all samples were centrifuged at 13,000 RPM for 10 min in a Labofuge 400 R centrifuge (Thermo Fisher Scientific, Waltham, MA, USA). IMUNOR components were separated on an Acquity UPLC Peptide BEH C18, 300 Å 1.7 µm (2.1 × 150 mm) column (Waters, Milford, MA, USA) [7] employing gradient mixing of 0.1% trifluoroacetic acid (TFA) (Merck, Darmstadt, Germany) aqueous solution with 0.1% TFA acetonitrile (Merck, Darmstadt, Germany) aqueous solution (3:7, *v*/*v*) at a flow rate of 0.4 mL/min. Chromatographic analysis was performed over 21 min when 100% aqueous TFA gradually decreased to 0% from the 4th to the 14th minute, then returned back to 100% from the 15th minute until the end of the analysis. The column temperature was kept at 35 °C whilst the temperature of autosampler was set to 8 °C. Eluted IMUNOR components were identified by diode array detector (DAD) allowing full spectral detection from 200 to 400 nm. Chromatographic data were analyzed by the OpenLab chromatography data system (Agilent Technologies, Santa Clara, CA, USA).

### 4.7. Size-Exclusion (SE) HPLC

Size-exclusion (SE) chromatography was employed to investigate the molecular weights of IMUNOR components. This method is considered as a characterization method according to the International Conference of Harmonization (ICH) [25]. IMUNOR Substance samples (10 mg sample in 2 mL purified water) were diluted in mobile phase in ratio 1:9 (*v*/*v*). Before injecting 3 μL of the sample solution into the 1260 Infinity II UHPLC system (Agilent Technologies, Santa Clara, CA, USA), all samples were centrifuged at 13,000 RPM for 10 min in a Labofuge 400 R centrifuge (Thermo Fisher Scientific, Waltham, MA, USA). IMUNOR components were separated on an Acquity UPLC Peptide BEH SEC, 125 Å 1.7 µm (4.6 × 150 mm) column (Waters, Milford, MA, USA) [5] which is suitable for analysis of peptides and proteins in the molecular weight range from 1 kDa–80 kDa. The column was calibrated using L-tryptophane (Agilent Technologies, Santa Clara, CA, USA) and Gel Filtration Standard mixture (Bio-Rad, Hercules, CA, USA) consisting of thyroglobulin, bovine γ-globulin, chicken ovalbumin, equine myoglobin, and vitamin B12, ranging from 0.2 kDa to 670 kDa. Phosphate buffer with 100 mM concentration and pH 7.0 (Merck, Darmstadt, Germany) was chosen as mobile phase at a flow rate of 0.4 mL/min. The column temperature was kept at 35 °C whilst the temperature of the autosampler was set to 8 °C. All IMUNOR components were eluted within 10 min and identified by diode array detector (DAD) allowing full spectral detection from 200 to 400 nm. Molecular weights of IMUNOR components were calculated by GPC/SEC Software for the OpenLab chromatography data system (Agilent Technologies, Santa Clara, CA, USA)) employing narrow standard calibration.

### 4.8. Amino Acid Analysis

We developed an RP HPLC method to determine the amino acid composition. The content of amino acids in IMUNOR samples was determined by acidic hydrolysis of 10 mg IMUNOR samples in 6 M HCl containing 1% of phenol (Merck, Darmstadt, Germany). IMUNOR was hydrolyzed for 24 h at 110 °C. Amino acids released from the IMUNOR components were further derivatized employing Advance Bio, an automated online pre-column derivatization kit (Agilent Technologies, Santa Clara, CA, USA). Hydrolyzed samples were resuspended by the HPLC injector in borate buffer and primary amino acids were derived from o-phthalaldehyde (OPA). The secondary amino acids were derived from 9-fluorenylmethyl chloroformate (FMOC); both reagents were added by the HPLC injector according to manufacturer’s instructions [32]. Derivatized samples were then injected into the 1200 Series HPLC system (Agilent Technologies, Santa Clara, CA, USA) and analyzed by RP chromatography on an AdvanceBio AAA C18 HPLC column (2.7 µm 4.6 × 100 mm) coupled with an AdvanceBio AAA guard column (2.7 µm, 4.6 × 5 mm) (Agilent Technologies, Santa Clara, CA, USA). Gradient elution at a 1.5 mL/min flow rate was applied according to the manufacturer’s instructions, and 10 mM borate-phosphate buffer (pH 9.2) (Merck, Darmstadt, Germany) with acetonitrile: methanol: water (45:45:10, *v*:*v*:*v*) mixture (Merck, Darmstadt, Germany) were used as mobile phase components. The temperatures for the column and autosampler were set to 40 °C and 8 °C, respectively. Separated amino acids were detected by a UV/VIS detector at 338 nm for OPA-derivatized primary amino acids and at 262 nm for FMOC-derivatized secondary amino acids. The determined amino acids were quantified by an external standard method using AdvanceBio amino acid standard solutions (Agilent Technologies, Santa Clara, CA, USA). Chromatographic data were analyzed by the OpenLab chromatography data system (Agilent Technologies, Santa Clara, CA, USA).

### 4.9. Data Analysis

In determining the homogeneity of protein or peptide components among IMUNOR batches, we calculated the mean and standard deviation for the protein quantification, and relative standard deviation (% RSD) for the chromatographic peaks in the HPLC analysis. In the amino acid composition assay, we also estimated the average relative abundance of proteinogenic amino acids and the standard error (SE) of the mean.

## 5. Conclusions

In this study, we performed an extensive physicochemical characterization of IMUNOR. For its evaluation, we utilized five to nine representative batches from a regular manufacturing process on an industrial scale produced at the GMP facility. We developed several techniques for such a physicochemical characterization by an application of a comprehensive approach to evaluate every possible aspect of its qualitative characteristics, including its complex starting material, which is porcine blood, with consecutive separation of leukocytes. 

This study confirmed that the active substance of IMUNOR is DLE. Furthermore, we confirmed that its manufacturing process is consistent and robust, proving high reproducibility among all tested batches in each/every qualitative parameter applied, such as total peptide content, DNA content, RP HPLC and SE HPLC profiles of the main peaks, amino acid composition, and SDS PAGE pattern.

Our further investigation of the product will focus on improving the SE-HPLC method and for the detailed identification of IMUNOR compounds. Additionally, the most ap-propriate analytical method for the quality control of IMUNOR will be selected and vali-dated. Moreover, LC-MS characterization of IMUNOR is currently being implemented, and based on the outcomes, the identified proteins or their residues will be investigated for their potential therapeutic effects.

## Data Availability

The original contributions presented in the study are included in the article, and further inquiries can be directed to the corresponding authors.
